# B-Site Fe/Re Cation-Ordering Control and Its Influence on the Magnetic Properties of Sr_2_FeReO_6_ Oxide Powders

**DOI:** 10.3390/nano12203640

**Published:** 2022-10-17

**Authors:** Zhuowei Wang, Qingkai Tang, Zhiwei Wu, Kang Yi, Jiayuan Gu, Xinhua Zhu

**Affiliations:** National Laboratory of Solid State Microstructures, School of Physics, Nanjing University, Nanjing 210093, China

**Keywords:** double-perovskite oxides, Sr_2_FeReO^6^ powders, sol–gel process, anti-site defects, magnetic properties, microstructural characterization

## Abstract

Double-perovskite oxide Sr_2_FeReO_6_ (SFRO) powders have promising applications in spintronics due to their half-metallicity and high Curie temperature. However, their magnetic properties suffer from the existence of anti-site defects (ASDs). Here, we report on the synthesis of SFRO powders by the sol–gel process. The B-site cationic ordering degree (*η*) and its influence on magnetic properties are investigated. The results demonstrate that the *η* value is well controlled by the annealing temperature, which is as high as 85% when annealing at 1100 °C. However, the annealing atmospheres (e.g., N_2_ or Ar) have little effect on the *η* value. At room temperature, the SFRO powders crystallize in a tetragonal crystal structure (space group *I*4/*m*). They have a relatively uniform morphology and the molar ratios of Sr, Fe, and Re elements are close to 2:1:1. XPS spectra identified that Sr, Fe, and Re elements presented as Sr^2+^, Fe^3+^, and Re^5+^ ions, respectively, and the O element presented as O^2-^. The SFRO samples annealed at 1100 °C in N_2_, exhibiting the highest saturation magnetization (*M*_S_ = 2.61 *μ*_B_/f.u. at 2 K), which was ascribed to their smallest ASD content (7.45%) with an anti-phase boundary-like morphology compared to those annealed at 1000 °C (ASDs = 10.7%) or 1200 °C (ASDs = 10.95%).

## 1. Introduction

With the research and development of charge-based semiconductor microelectronics, the feature sizes of electronic devices continue to shrink and approach physical limits, owing to the quantum effects of electrons [1,2,3]. To solve the issue of the bottleneck of Moore’s law, several novel alternative methodologies (e.g., nanoelectronics, spintronics, and quantum information technologies) have recently been proposed [4,5,6]. Among the possible potential methodologies, spintronics operated on the electronic spin degree is considered to be the technology with the most potential due to its good compatibility with conventional semiconductor electronics. As a result, many technologies that are used in recurrent charge-based semiconductor electronics can be easily transferred to spintronics [2]. To realize the practical spintronic devices operating at room temperature (RT), it is urgent to find the suitable materials that exhibit large spin polarizability and a high magnetic Curie temperature *T*_C_ (well above RT), which is the main issue in this technological breakthrough [7,8]. Much effort has been devoted to this issue in the past decade. It has been found that A_2_B′B″O_6_ double-perovskite (DP) oxides have a high spin polarization and large *T*_C_ values well above RT, and they have become the promising candidate for RT spintronic devices [9]. Kobayashi et al. first reported the ordered Sr_2_FeMoO_6_ (SFMO) DP oxides exhibiting a high magnetoresistance (MR) (MR = 10% at 300 K and 7 T) with *T*_C_ = 412 K (much higher than RT) [7]. Theoretical band calculations also predicted their half-metallicity with saturation magnetization (*M*_S_) of 4.0 *μ*_B_/f.u. (formula unit) [10,11]. The half-metallicity originates from the itinerant 4*d*^1^ electron of the Mo^5+^ ion [7]. Because Sr_2_FeReO_6_ (SFRO) DP oxides have an analogous crystal structure to the SFMO DP oxides, they should also exhibit similar magnetic properties, as confirmed by theoretical band calculations [12,13]. The crystal structure of these DP oxides is composed of corner-sharing B′O_6_ and B″O_6_ octahedra, which are regularly and alternatively arranged in the three crystallographic directions of DP oxides. The large A cations are located at the voids between these octahedra. Actually, anti-site defects (ASDs) at B-sites often appear in these DP oxides, which play an important role in spin polarizations (*P*) and *M*_S_ values [14,15,16]. The ordering degree (*η*) of the B-site cations in DP oxides are closely related to their synthesized conditions [17,18,19,20]. Generally, a higher cationic ordering degree can be achieved under higher synthesis temperatures [17] or a longer treatment time [20]. To date, several methods, such as conventional solid-state reaction [21], high-pressure method [22], molten salt synthesis [23,24], and wet-chemical routes [22,25,26], have been used to synthesize SFRO DP oxide powders. However, the synthesis of high-quality SFRO DP powders is still challenging work, especially for achieving a high ordering degree of Fe/Re at B-sites. This is an essential prerequisite for their desirable magnetic properties. At present, this issue is a major barrier in the development of SFRO DP oxides for practical use in spintronics.

In this work, SFRO DP powders were synthesized by the sol–gel process, followed by annealing at different temperatures (800–1200 °C) and gas atmospheres (N_2_ and Ar gases). The B-site Fe/Re cation-ordering control and its influence on the magnetic properties of SFRO DP powders were explored. The present results shed light on a deeper understanding of the processing–structure–property relationships in the SFRO DP oxide system, which has great potential for applications in the fields of electronic storage devices and spintronic devices.

## 2. Materials and Methods

### 2.1. Synthesis of SFRO DP Powders

In this work, the SFRO DP powders were synthesized by the sol–gel process. The starting materials were strontium carbonate (SrCO_3_), iron nitrate (Fe(NO_3_)_3_·9H_2_O), metal rhenium (Re), rhenium oxide (Re_2_O_7_), citric acid (CA), ethylene glycol (EG), and nitric acid (HNO_3_). They are purchased from Sinopharm Chemical Reagent Co., Ltd., Shanghai, China. First, stochiometric amounts of the raw materials were weighed in a molar ratio of Sr:Fe:Re:O = 2:1:1:6., and the molar ratio of (Sr^2+^+Fe^3+^+Re^5+^):CA: EG was kept as 1:4:3. Specifically, 2.9904 g SrCO_3_, 4.0278 g Fe(NO_3_)_3_·9H_2_O, 0.3088 g Re, 2.0156 g Re_2_O_7_, 8.4123 g CA, and 1.7560 g EG were dissolved in an aqueous citric acid solution and well mixed by a magnetic stirrer. After continuous stirring at 100 °C for 5 h, a yellowish-brown clear solution was obtained. This solution was transferred into an alumina crucible and heated up to 200 °C in a muffle furnace for 12 h, and then naturally cooled to room temperature. Subsequently, dry brown homogeneous precursors were obtained, which were ground into powders by an agate mortar and pestle. The brown powders were annealed at different temperatures (800–1200 °C) for 12 h in N_2_ (or Ar) atmospheres, and then naturally cooled down to 25 °C. Finally, the black crystallized SFRO powders were obtained.

### 2.2. Structural and Physical Characterization

The phase structure of the SFRO DP powders was examined by powder X-ray diffraction carried out at RT using a diffractometer (XRD, Bruker D8 Advance, Karlsruhe, Germany) under Cu Kα radiation (*λ* = 1.5406 Å). The XRD data were collected from 2*θ* = 10° to 80° with a step of 0.02° and a count time of 10 s per step. Refined structural parameters were obtained from the Rietveld refinements of the experimental XRD data using the FullProf computer program package [27]. During refinement, a pseudo-Voigt function was used to generate the line shape of the diffraction peaks. The unit cell lattice parameters and phase fractions, scale factor, background, atomic coordinates, and isothermal temperature parameters (U_iso_) were refined successfully. Three major parameters, such as profile (R_p_), weighted profile factors (R_wp_), and goodness of fit (χ^2^), were used to define the fitting quality of the theoretically generated pattern compared to the experimental one [28]. Scanning electron microscopy (SEM, S-3400N II, Hitachi, Tokyo, Japan) was used to examine the surface morphology of the SFRO DP powders. Their chemical compositions were determined from energy-dispersive X-ray spectroscopy (EDS) data collected in a mapping mode. The chemical valence states of the constituent elements in the SFRO DP powders and their binding energies (BE) were determined by X-ray photoelectron spectroscopy (XPS) spectra, which were collected from a PHI5600 ESCA 120 (ULVAC PHI) system with an Al Kα excitation source. The isothermal magnetization curves of the SFRO powder samples were measured at 2 K and 300 K by a commercial superconducting quantum interference device (SQUID; Quantum Design MPMS XL-7, San Diego, CA, USA) magnetometer with respect to magnetic fields up to 6.5 T. 

## 3. Results

### 3.1. XRD Analysis and Its Rietveld Refinement

**Figure 1:**
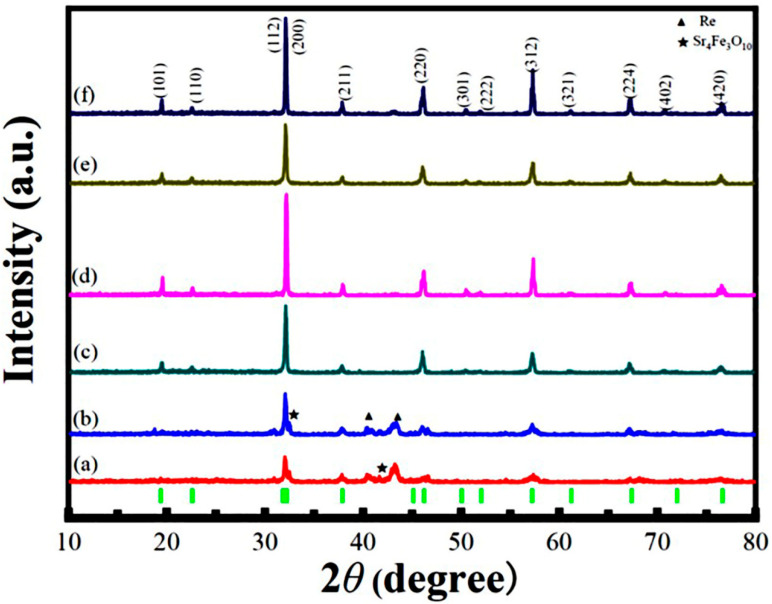
Room temperature XRD patterns of the SFRO powders annealed for 12 h at different temperatures and atmospheres. (**a**) 800 °C, (**b**) 900 °C, (**c**) 1000 °C, (**d**) 1100 °C under N_2_ atmosphere, (**e**) 1100 °C under Ar atmosphere, and (**f**) 1200 °C under N_2_ atmosphere. The green tick marks represent the positions of the allowed Bragg reflections.

RT XRD patterns of the SFRO powders annealed at different temperatures and atmospheres are shown in Figure 1, which can be well indexed in a tetragonal unit cell with a *I*4*/m* space group. It was found that the SFRO powders annealed at low temperatures (e.g., 800 °C and 900 °C, curves a and b) in N_2_ atmospheres were associated with some impure phases of Sr_4_Fe_3_O_10_ and metal Re, but they almost disappeared in the samples annealed at 1000 °C (curve c). By further increasing the annealing temperature up to 1100 °C (curve d), the SFRO powders were completely free of these impure phases, and the (101) superstructural reflection, reflecting the long-range Fe/Re ordering, had a much stronger intensity. As the annealing temperature increased up to 1200 °C (curve f), the peak intensity of the (101) superstructural reflection reduced. In the literature, the intensity ratio of the (101) superstructural diffraction peak (appearing around 2θ ≈ 19°) to the (112) and (200) diffraction peaks (around 2θ ≈ 32°) is defined as *R* = *I*_101_/(*I*_112_ + *I*_200_), which is used to estimate the *η* value in DP oxides. The *η* value is evaluated by the subsequent equation [17]:

(1) $$\eta =\sqrt{\frac{R_{\exp}}{R_{\mathrm{theo}}}}$$

where *R*_exp_ and *R*_theo_ represent the experimental and theoretical intensity ratios of the (101) to the (112) and (200) diffraction peaks, respectively. The experimental *R*_exp_ values were obtained from experimental XRD patterns, and the theoretical *R*_theo_ value (=25.1%) was reported by Jain et al. [29]. Therefore, the *η* values of the SFRO powders annealed at 1000, 1100, and 1200 °C in N_2_ atmospheres were estimated to be 78.6%, 85.1%, and 78.1%, respectively. It has been reported that the *η* value (B-site ordering degree) is related to the ASD content in SFRO powders, which is described by the following equation [30]:

*η* = 1 − 2∗ASDs (2)

Therefore, the ASD contents in SFRO powders annealed at 1000, 1100, and 1200 °C in N_2_ atmospheres were calculated to be 10.7%, 7.45%, and 10.95%, respectively. This indicates that the SFRO powders annealed at 1100 °C in N_2_ atmospheres exhibit the highest cationic ordering degree at the B-site. To investigate the influence of the annealing atmosphere on the ordering degree of B-site ions in SFRO powders, two samples annealed at the same temperature (1100 °C), but under different atmospheres (N_2_ vs. Ar), were comparatively investigated. Their XRD patterns are demonstrated in Figure 1d,e, respectively. The *η* value of the SFRO powders annealed at 1100 °C under Ar atmospheres was determined to be 85.4%, and the corresponding ASD content was 7.30%. This indicates that the annealing atmosphere has little influence on the *η* values of the SFRO powders annealed under different atmospheres. Based on the above analyses of the XRD data, the optimized annealing conditions for the SFRO powders synthesized by sol–gel process were obtained, which were annealing at 1100 °C under N_2_ or Ar atmospheres. Considering the economic cost, in the following discussion, we focus on the SFRO powders annealed at 1000, 1100, and 1200 °C in N_2_ atmospheres and annealed at 1100 °C in Ar atmospheres, which are denoted as samples A-D, respectively. Previously, Retuerto et al. [22] synthesized the SFRO powders by a soft-chemistry procedure, where the precursors were heated to 1200 °C for 4 h and to 1400 °C for 1.5 h under N_2_ atmospheres. Their SFRO powder samples exhibited *η =* 75%. Recently, Leng et al. [25] also synthesized SFRO powders by a sol–gel process, followed by annealing at 1000 °C for 24 h in Ar atmospheres. The SFRO samples had *η =* 67.5% and *M*_S_ = 2.0 *μ*_B_/f.u. at 2 K. Blanco et al. [26] reported on the synthesis of SFRO powders by the soft-chemistry method, where SFRO powders were synthesized from nanoscale precursors prepared by the wet-chemical route using citric acid. In addition, to obtain the SFRO powders with a high purity, two thermal annealing processes were used. Finally, the obtained SFRO powders possessed *η =* 75.5%. 

**Figure 2:**
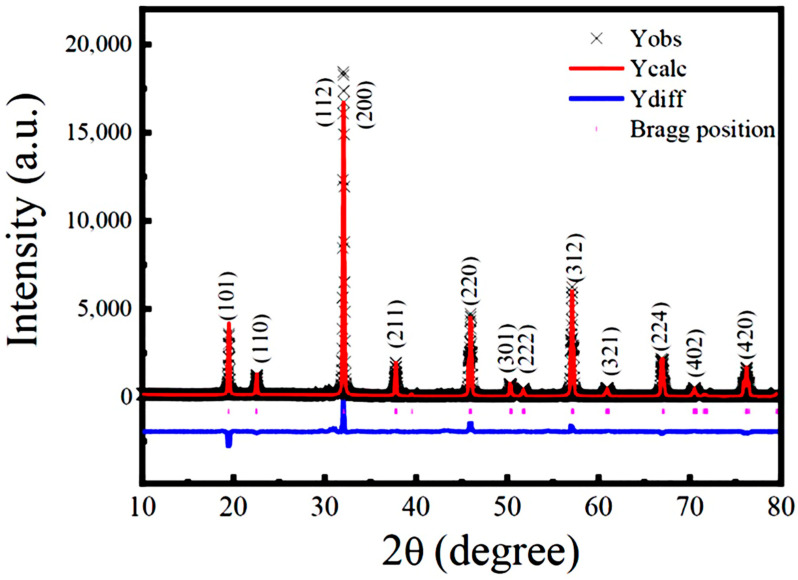
Rietveld refinements on the XRD data of the SFRO powders (sample B) annealed at 1100 °C under N_2_ atmosphere. Black cross symbols present the experimental data, the red solid line indicates the simulated XRD profile, and the tick marks represent the positions of the allowed Bragg reflections. The bottom continuous blue line stands for the difference between the experimental and theoretical patterns.

To obtain the structural parameters, Rietveld refinement on the experimental XRD data of sample B (SFRO powders annealed at 1100 °C under N_2_ atmospheres) was performed. The refined XRD pattern is shown in Figure 2, where the red solid line indicates the simulated diffractogram, the black cross symbols correspond to the experimental data, the blue line represents the difference between the experimental and theoretical patterns, and the green line refers to the background. The short vertical lines represent the positions of the allowed Bragg reflections. All diffraction peaks can be well indexed in a *I*4*/m* tetragonal structure without any detected impurities. The quality of Rietveld refinement in this work was evaluated by fitting parameters, such as the chi-square factor (*χ*^2^), profile (*R*_p_), and weighted profile factor (*R*_wp_), which were obtained as *χ*^2^ = 0.8665, *R*_p_ = 8.59%, and *R*_wp_ = 12.66%. The refined lattice parameters, selected bond length and bond angle, and the fitting parameters are presented in Table 1. These values match well with the data reported in the literature [22,25,26,31,32,33,34]. 

### 3.2. SEM Observation and EDS Analysis

**Figure 3:**
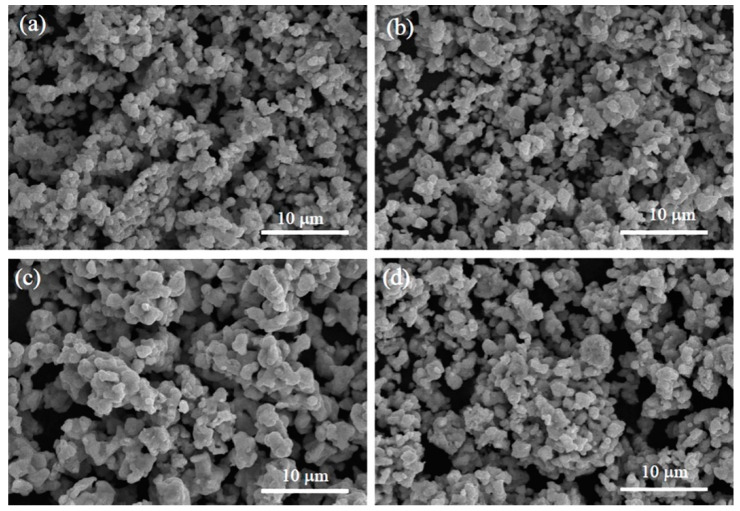
SEM images of samples A–D. (**a**) Sample A, (**b**) sample B, (**c**) sample C, and (**d**) sample D.

**Figure 4:**
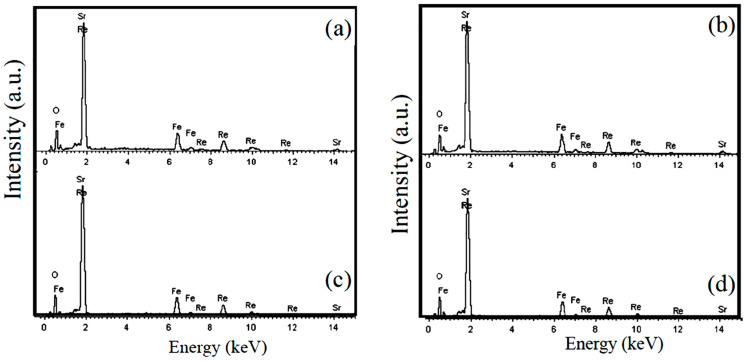
EDS spectra collected in a mapping mode for samples A–D. (**a**) sample A, (**b**) sample B, (**c**) sample C, and (**d**) sample D.

Figure 3a–d shows the SEM images of samples A–D, respectively. All the SEM images reveal homogeneous distributions of aggregated block-like particles with polyhedral morphology. As shown in Figure 3a–c, the average particle size ($\bar{D}$) increased from 0.85 μm to 1.40 μm as the annealing temperature increased from 1000 °C to 1200 °C in N_2_ atmospheres, but the $\bar{D}$ value had no apparent change between sample B ($\bar{D}$ = 0.95 μm) and sample D ($\bar{D}$ = 0.96 μm) annealed under different N_2_ and Ar atmospheres, respectively. Details are shown in Figure 3b,d, respectively. The EDX spectra for the scanned areas of samples A–D are demonstrated in Figure 4, where the signals of the component elements (Sr, Fe, Re, and O) can be clearly identified, and no trace of impurity elements is observed. This confirms the purity of the elaborated samples. The average chemical composition of the SFRO powders was determined to be Sr_2.03_Fe_1.04_Re_1.03_O_5.91_ from the EDS data, very close to the nominal stoichiometric composition of Sr_2_FeReO_6_. 

### 3.3. XPS Spectra Analysis

**Figure 5:**
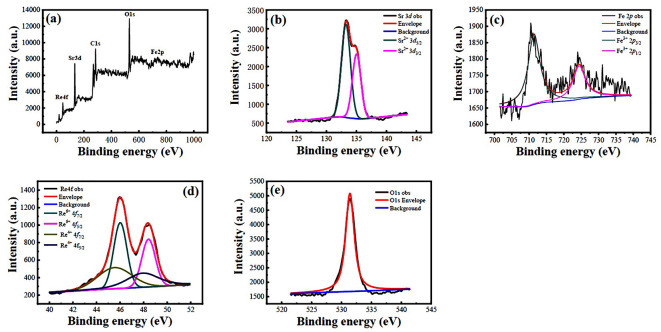
XPS spectra of sample A. (**a**) Survey scan XPS spectrum, (**b**–**e**) regional scan Sr 3d, Fe 2*p*, Re 4*f*, and O 1s XPS spectra, respectively.

The chemical valence states of the constituent elements in samples A–D were investigated by XPS spectra. Figure 5a shows a survey scan XPS spectrum of sample A with BE in the range of 0–1000 eV, where the Re 4*f*, Sr 3*d*, Fe 2*p*, and O 1*s* XPS peaks are clearly observed, confirming the existence of Sr, Fe, Re, and O elements in sample A. In addition, the C1*s* XPS peak with a BE value of 284.60 eV was also observed due to the SFRO powders attached to the adhesive carbon tape during XPS measurements. The regional scan of Sr 3*d* XPS spectrum is shown in Figure 5b, which was split into two peaks of Sr 3*d*_5/2_ and Sr 3*d*_3/2_ because of the spin–orbit coupling. The two split peaks were located at BE values of 132.90 eV and 135.10 eV, respectively, indicating the chemical valence state of Sr element in +2 in sample A [35]. The local scans for Fe 2*p* and Re 4*f* XPS spectra are displayed in Figure 5c,d, respectively. As shown in Figure 5c, the Fe 2*p* XPS spectrum was also split into two sub-peaks with BE values of 710.5 eV (Fe 2p_3/2_) and 724.5 eV (Fe 2p_1/2_), respectively, which is contributed from the spin–orbit coupling. These BE values are characteristics of the Fe^3+^ species, indicating the existence of Fe^3+^ ions in sample A. This matches well with the data reported by the references [36,37]. As shown in Figure 5d, two separated Re 4*f*_7/2_ and 4*f*_5/2_ XPS peaks with a BE energy difference of 2.4 eV were observed, which are the characteristics of 4*f* doublet, owing to the spin–orbit coupling. To make a well fitting of the Re 4*f* XPS peaks, two doublet XPS peaks were utilized with BE energies of 45.2 eV and 47.6 eV, and of 46.0 eV and 48.4 eV. The first two components were assigned to the Re^4+^ species while the latter two ones were assigned to the Re^6+^ species [35]. The average chemical oxide state of the Re element was determined to be +5, based on the deconvoluted XPS peak areas. It was observed that the local scan for the O 1*s* XPS spectrum shown in Figure 5e only displayed one peak, located at a BE value of 531.1 eV, which corresponds to the lattice oxygen in sample A [38,39]. Similar XPS spectra of samples B-D were also obtained, which are demonstrated in Figure S1–S3, respectively. The present XPS spectra identified that the Sr element was present as Sr^2+^, and Fe and Re elements exist in the form of Fe^3+^ and Re^5+^ ions, while oxygen was present as lattice oxygen (O^2−^). 

### 3.4. Magnetic Properties

**Figure 6:**
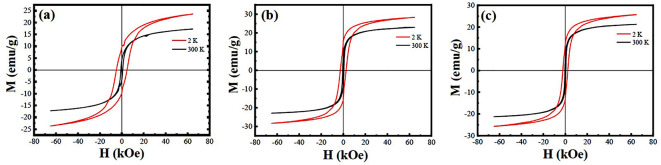
*M-H* hysteresis loops of samples A–C measured at 2 K and 300 K, respectively. (**a**) Sample A, (**b**) sample B, (**c**) sample C.

Figure 6 shows the *M-H* hysteresis loops of samples A–C measured at 2 K and 300 K, respectively. They exhibit the *M-H* hysteresis loops, indicating ferromagnetic behaviors. It was also noticed that the magnetization (*M*) increased nonlinearly with the increase in the external magnetic fiel, which almost achieved the saturation state under a magnetic field of 65 kOe. Their *M*_S_, remanent magnetization (*M*_r_), and coercive field (*H*_c_) measured at 2 K and 300 K are presented in Table 2. It was found that sample B exhibits the highest *M*_S_ and *H*_c_ values due to its highest Fe/Re ordering degree. The *M*_S_ value of 2.61 *μ*_B_/f.u. at 2 K for sample B was much higher than those reported for the SFRO powders synthesized by wet-chemical routes [22,25]. The ferromagnetism of the SFRO compound can be understood from the AFM coupling between Fe^3+^ ion (3*d*^5^: $t_{2g}^{3}e_{g}^{2},\;m_{\mathrm{Fe}}=5\;\mu_{B}$) and Re^5+^ ion (5*d*^2^: t2g2,mRe=2 μB) via the Fe^3+^-O(2*p*)-Re^5+^ magnetic paths. The theoretical *M*_S_ value for the SFRO compound is expected to be 3.0 *μ*_B_/f.u. by the assumption of the collinear FM configuration [Fe^3+^(5*μ*_B_)][Re^5+^(2*μ*_B_)] or [Fe^2+^(4*μ*_B_)][Re^6+^(1*μ*_B_)]. Due to the existence of ASDs in the SFRO samples, their experimental *M*_S_ value was reduced. Supposing the ASD content is x, and only considering the spin-only contribution, the Fe sublattice (B′-site) is occupied by (1−x) Fe ions and x Re ions, and the Re sublattice (B″-site) is occupied by (1−x) Re ions and x Fe ions, then the experiment *M*_S_ can be calculated by the following equation:

(3) Msexp=(1−x)mFe+xmRe−[xmFe+(1−x)mRe]=(1−2x)(mFe−mRe)=(1−2x)Mscal

where $M_{s}^{\exp}$ is the experimental saturation magnetization, $M_{s}^{\mathrm{cal}}$ is the theoretical saturation magnetization (m_Fe_ − m_Re_), m_Fe_, and m_Re_ are the magnetic moments of Fe^3+^ and Re^5+^ ions, respectively. From Equation (3), the ASD content (x) can be expressed as

(4) $$ASD\;\left(x\right)=\frac{\left(1-\frac{M_{s}^{exp}}{M_{s}^{cal}}\right)}{2}$$

By using Equation (4), the ASD content (x) in samples A–C could be calculated as 13.85%, 6.50%, and 10.5%, respectively. The corresponding *η* values (Fe/Re ordering degrees at B-site) were determined to be 72.3%, 87.0%, and 79.0%, respectively, which were comparable with data obtained from the experimental XRD results. Details can be seen in Table 2. In the SFRO samples with low levels of Fe/Re cationic ordering, ASDs were generated in the form of Fe-on-Re (Fe_Re_) and Re-on-Fe (Re_Fe_), respectively, which are dependent upon the prepared conditions. The formation of ASDs led to a reduction in the magnetization of the SFRO oxides via a strong AFM coupling between the ASDs and the host atoms [40]. Theoretical calculations have demonstrated that the formation energies of isolated ASDs, such as Fe_Re_ and Re_Fe_, in SFRO are less than 1.0 eV; thus, they are likely to appear as dominant defects [41]. The two species of isolated ASDs favor to form a cluster, Fe_Re_-Re_Fe_ (1NN interatomic distance), due to a low formation energy of ~0.6 eV. The BE value for a single Fe_Re_-Re_Fe_ pair cluster was calculated to be 0.12 eV in SFRO, and the positive BE value indicates the formation of such a single cluster was endothermic. In the present work, samples A–C were annealed at 1000 °C, 1100 °C, and 1200 °C in N_2_ atmospheres, respectively, and the corresponding thermal energies (*k*_B_*T*) were equal to 0.110 eV, 0.118 eV, and 0.127 eV, respectively. Therefore, the Fe_Re_-Re_Fe_ pair clusters in sample B were readily formed due to the thermal energy, much closer to the BE value of a single Fe_Re_-Re_Fe_ pair cluster. Hence, the two pairs may like to be close to each other due to their attractive interaction, and the ASDs are clustered in the form of an anti-phase-boundary (APB)-like feature in the SFRO compound, which separates two adjacent anti-phase domains (APDs) with a revised Fe/Re arrangement. Each APD is perfectly ordered, whereas the APBs are composed of Fe-O-Fe or Re-O-Re bonds. This picture has been confirmed by direct transmission electron microscopy (TEM) observations in the Re-excess SFRO crystal [40]. Similar TEM results were also reported in A_2_FeMoO_6_ (A = Sr, Ba) and Sr_2_CrReO_6_ compounds [42,43,44,45]. Recently, Yuan et al. reported a robust long-range magnetic correlation across APBs in Sr_2_CrReO_6_ thin films by using a resonant elastic X-ray scattering technique [46]. They found that the structural correlation length of the Sr_2_CrReO_6_ thin films was limited by the size of the APDs of about 15 nm, while the magnetic correlation length was at least 90 nm, much larger than the average domain size. The two neighboring APDs were antiferromagnetically coupled. The existence of APDs with a magnetic correlation in DP oxides has been argued to enhance their MR values because of the cooperative spin rotation between different domains [47]. The largest *M*_S_ value of 2.61 *μ*_B_/f.u. (at 2 K) was observed in sample B, which could be ascribed to the existence of clustered ASDs in the form of APB (a planar defect) and the highest Fe/Re ordering degree at the B-site. Of course, the Re vacancy (V_Re_) is also considered to be a factor that influences the magnetic properties of SFRO oxide because V_Re_ is likely to be formed in the oxides. However, first principles calculations have demonstrated that the formation energy of V_Re_ is about 4.0 eV in SFRO oxide [41], which is much higher than the thermal energy provided by the annealing temperature. Therefore, the contribution to the magnetic properties of SFRO powders from V_Re_ is negligible. 

## 4. Conclusions

Double-perovskite SFRO powders were synthesized by the sol–gel process, followed by annealing at different temperatures under atmospheres of N_2_ and Ar gases. It was found that the cation-ordering degree can be well controlled by the annealing temperature, whereas the annealing atmosphere has little effect on the cation-ordering degree. The SFRO powders annealed at 1100 °C in N_2_ atmospheres had the highest *η* value of 85.1% and the largest *M*_S_ value of 2.61 *μ*_B_ /f.u. at 2 K. The corresponding coercive magnetic field (*H*_C_) was 15596 Oe. XRD patterns and their Rietveld refinements revealed that the SFRO powders crystallize in a tetragonal crystal structure (*I*4/*m* space group) at room temperature. The refined lattice parameters were *a* = 5.56329(1) Å, *c* = 7.89942(2) Å, and had a unit cell volume of 244.49(1) Å^3^. SEM images showed that the SFRO particles are relatively uniform with a polyhedral morphology. Their mean particle size increased with an increase in annealing temperature. The EDS data identified the atomic ratios of Sr:Fe:Re, approaching the nominal stoichiometric value. XPS spectra confirmed that the Sr, Fe, Re, and O elements in SFRO powders are present as Sr^2+^, Fe^3+^, Re^5+^, and O^2-^, respectively. The highest *M*_S_ value observed in the SFRO powders annealed at 1100 °C under N_2_ gas can be ascribed to the existence of the lowest content of ASDs (ASDs = 7.45%) with an APB-like morphology, as compared with the samples annealed at 1000 °C with ASDs = 10.7%), or at 1200 °C with ASDs = 10.95% under N_2_ gas. The present work offers an effective route to controlling the B-site Fe/Re cation ordering, and subsequently modifying the magnetic properties of SFRO powders.

## Figures and Tables

**Table 1:** Refined lattice parameters, selected bond length and bond angles, and the fitting parameters of the SFRO powders annealed at 1100 °C under N_2_ atmosphere.

Sample	Lattice Parameters	Space Group	Average Bond Length (Å)		Average Bond Angles (◦)		Fitting Parameters
SFRO	Tetragonal structure *a* = *b* = 5.57677(1) Å *c* = 7.91916(1) Å *V* = 246.29(2) Å^3^	*I*4/*m*	<Fe-O_1_>	1.99196(18)	<Fe1-O_1_-Re1>	170.146(0)	*χ*^2^ = 0.8665 *R*_p_ =8.59% *R*_wp_ = 12.66%
			<Fe-O_2_>	1.99642(18)			
			<Re-O_1_>	1.96603(17)	<F1e-O_2_-Re1>	180.000(0)	
			<Re-O_2_>	1.96316(17)			

**Table 2:** Magnetic properties of samples A–C and their Fe/Re ordering degrees (*η*), and the ASD contents deduced from the XRD data and magnetic data, respectively.

Sample	2 K			300 K			*η* Value from XRD Data	ASD Content from XRD Data	*η* Value from Magnetic Data	ASD Content from Magnetic Data
	$M_{s}^{exp}$ $\left(\mu_{B}/f.u.\right)$	*M*_r_ (*μ*_B_/f.u.)	*H*_c_ (Oe)	$M_{s}^{exp}$ $\left(\mu_{B}/f.u.\right)$	*M*_r_ (*μ*_B_/f.u.)	*H*_c_ (Oe)				
A	2.17	0.90	9757	1.59	0.38	4107	78.6%	10.70%	72.3%	13.85%
B	2.61	1.43	15,596	2.11	0.74	8001	85.1%	7.45%	87.0%	6.50%
C	2.37	1.19	12,925	1.96	0.63	6531	78.1%	10.95%	79.0%	10.50%

## Data Availability

The data presented in this work are available on request from the corresponding author. The data are not publicly available due to privacy.

## Supplementary Materials

The following supporting information can be downloaded at: https://www.mdpi.com/article/10.3390/nano12203640/s1, Figure S1: XPS spectra of the sample B. (a) Survey scan XPS spectrum, (b)–(e) regional scan Sr 3d, Fe 2p, Re 4f, and O 1s XPS spectra, respectively; Figure S2: XPS spectra of the sample C. (a) Survey scan XPS spectrum, (b)–(e) regional scan Sr 3d, Fe 2p, Re 4f, and O 1s XPS spectra, respectively; Figure S3: XPS spectra of the sample D. (a) Survey scan XPS spectrum, (b)–(e) regional scan Sr 3d, Fe 2p, Re 4f, and O 1s XPS spectra, respectively.
