# Supplementary material for: B-Site Fe/Re Cation-Ordering Control and Its Influence on the Magnetic Properties of Sr2FeReO6 Oxide Powders
Source: Nanomaterials (Basel). 2022 Oct 17;12(20):3640. doi: 10.3390/nano12203640 (PMC9611336; doi:10.3390/nano12203640)
Supplement: Supplementary file 1 [file nanomaterials-12-03640-s001.zip › nanomaterials-1969075-supplementary.pdf]

nanomaterials

MDPI

Article

Supplemental Materials

B-Site Fe/Re Cation-Ordering Control and Its Influence on the Magnetic Properties of Sr_2FeReO_6 Oxide Powders

Zhuowei Wang, Qingkai Tang, Zhiwei Wu, Kang Yi, Jiayuan Gu and Xinhua Zhu *

National Laboratory of Solid State Microstructures, School of Physics, Nanjing University, Nanjing 210093, China

*Correspondence: xhzhu@nju.edu.cn

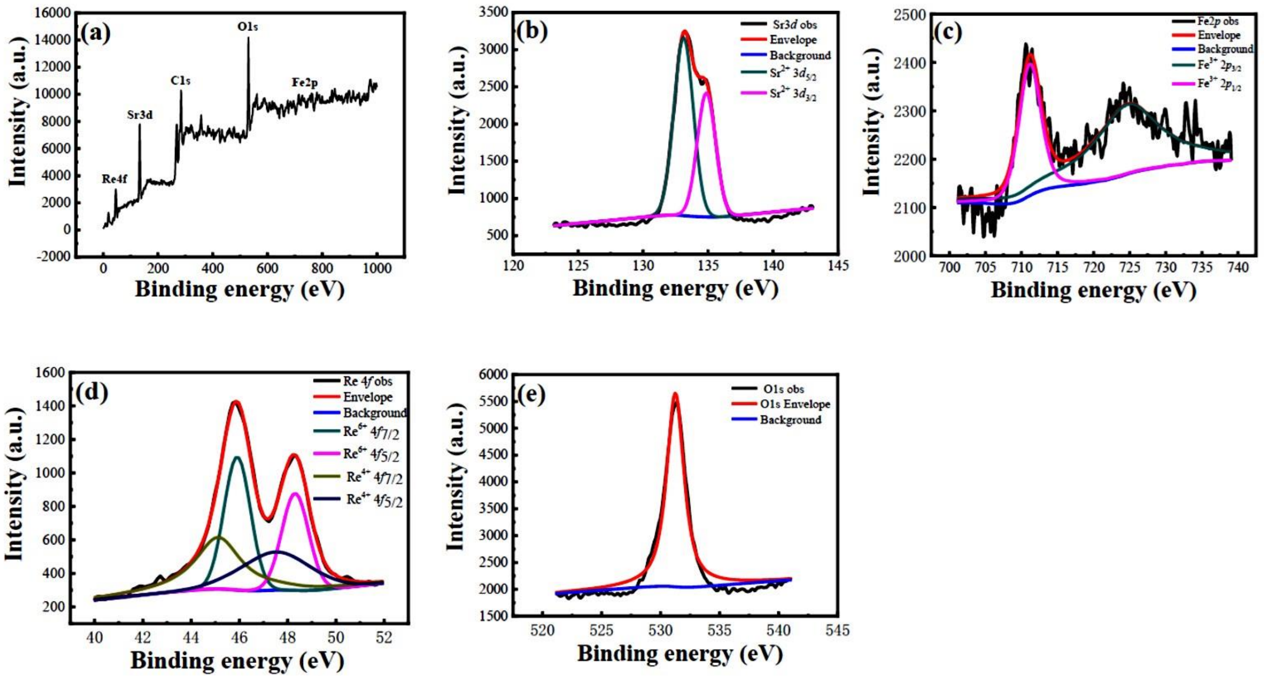

Figure S1. XPS spectra of the sample B. (**a**) Survey scan XPS spectrum, (**b**)-(**e**) regional scan Sr 3d, Fe 2*p*, Re 4*f*, and O 1s XPS spectra, respectively.

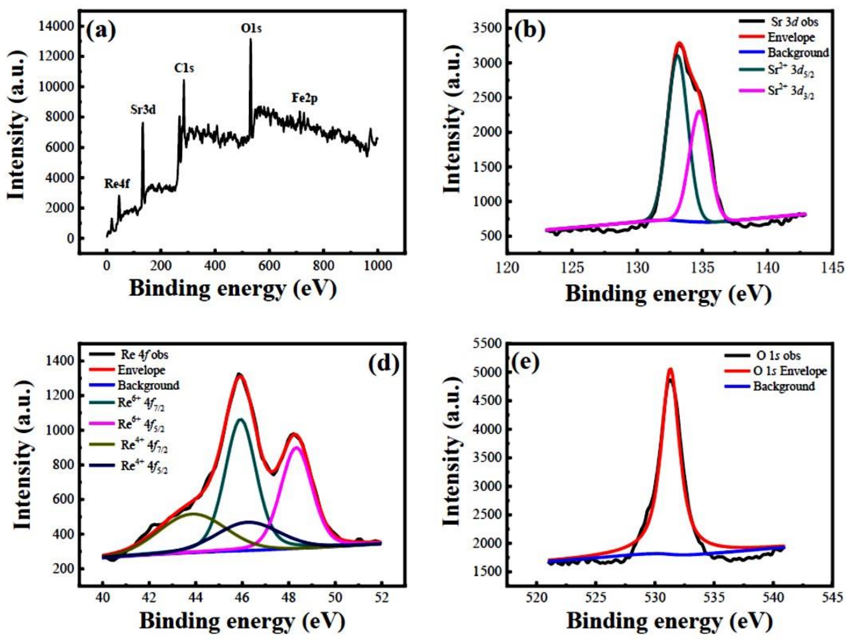

Figure S2. XPS spectra of the sample C. (**a**) Survey scan XPS spectrum, (**b**)-(**e**) regional scan Sr 3d, Fe 2*p*, Re 4*f*, and O 1s XPS spectra, respectively.

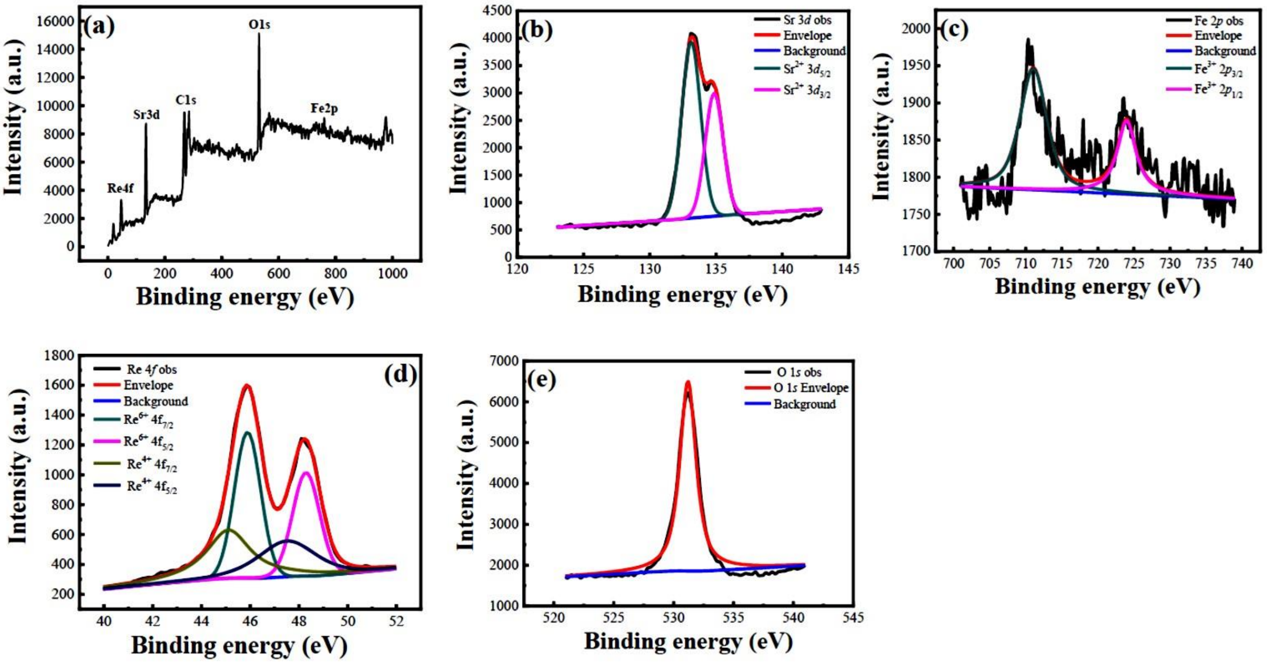

Figure S3. XPS spectra of the sample D. (**a**) Survey scan XPS spectrum, (**b**)-(**e**) regional scan Sr 3d, Fe 2*p*, Re 4*f*, and O 1s XPS spectra, respectively.
